# Tumor stem cell-derived exosomal microRNA-17-5p inhibits anti-tumor immunity in colorectal cancer via targeting SPOP and overexpressing PD-L1

**DOI:** 10.1038/s41420-022-00919-4

**Published:** 2022-04-23

**Authors:** Wei Sun, Junpeng Cui, Yang Ge, Jinshi Wang, Yifan Yu, Bing Han, Baolin Liu

**Affiliations:** The Sixth Department of General Surgery, Shengjing Hospital of China Medical University, 110004 Shengyang, Liaoning China

**Keywords:** Cancer, Diseases

## Abstract

Exosomes are known to transmit microRNAs (miRNAs) to affect human cancer progression, and miR-17-5p has been manifested to exert facilitated effects on colorectal cancer (CRC) progression, while the role of tumor stem cells-derived exosomal miR-17-5p in CRC remains unknown. We aim to explore the effect of CRC stem cells-derived exosomes (CRCSC-exos) conveying miR-17-5p on CRC. The exosomes were isolated from CRC stem cells and identified. HCT116 cells were transfected with speckle-type POZ protein (SPOP) interfering vector or co-cultured with exosomes carrying miR-17-5p mimic/inhibitor. Then, the proliferation, migration, invasion, and apoptosis of the cells were determined. The xenograft mouse model was constructed using BALB/C mice and the serum levels of T cell cytokines were assessed. Expression of miR-17-5p, SPOP, CD4, CD8 and programmed death ligand 1 (PD-L1) was detected. The targeting relationship between miR-17-5p and SPOP was verified. MiR-17-5p was upregulated and SPOP was downregulated in CRC tissues. CRCSC-exos transmitted miR-17-5p to HCT116 cells to promote malignant behaviors and suppress anti-tumor immunity of HCT116 cells. The overexpressed SPOP exerted opposite effects. SPOP was confirmed as a target gene of miR-17-5p. Upregulated CRCSC-exosomal miR-17-5p inhibits SPOP to promote tumor cell growth and dampen anti-tumor immunity in CRC through promoting PD-L1.

## Introduction

Colorectal cancer (CRC) kills almost 700,000 people every year, ranking the top four (after lung, liver, and stomach cancer) for inducing cancer-related death globally [1, 2]. CRC is a complex process featured by multiple genomic variations and the abnormal biological microenvironment [3]. About 55% of CRC cases happened in developed countries, indicating that it is more prevalent in developed regions [4]. The underlying mechanisms of CRC are multifactorial and the risk factors of CRC contain gender, age, and lifestyle, with underlying genetics significant to a lesser extent [5]. Although addressability, awareness, and screening have been developed, there remains 25% of the cases diagnosed as CRC at advanced stages [6]. Nowadays, CRC patients are treated with surgical resection, chemotherapeutic drugs, or adjuvant. These therapeutic strategies improved the overall survival rate of CRC patients. Nevertheless, the treatments were accompanied by severe side effects, such as weight loss, vomiting, nausea, and risk of infectious complications [7]. Thus, developing more effective strategies for the treatment of CRC is vital.

Exosomes are vesicles with 40–150 nm in diameter and released from several mammalian cell types. Exosomes can dock and fuse to the target cell membrane, transmitting exosomal surface proteins and cytoplasm. Thus, exosomes were usually used as delivery vehicles for microRNAs (miRNAs) as exosomes do not induce adverse immune responses and have a low risk for tumorigenesis [8]. MiRNAs are a type of small and single‑stranded RNA with ~22 nt in length and lack protein‑coding ability. miRNAs can regulate gene expression at a post‑translational level by binding to the 3’‑untranslated region (UTR) of target mRNAs, resulting in mRNA degradation or translation inhibition [9]. It has been reported that miR-96 promoted the occurrence and progression of CRC [10], and a study has revealed that the elevation of miR-224 promoted CRC cell migration and invasion [11]. The miR-17-5p–92 cluster was initially described as an oncogene cluster and identified to drive vital physiological responses during disease development [12]. In this cluster, miR-17-5p is specifically oncogenic and usually upregulated in human cancers [13, 14]. For instance, in the non-invasive measurement of CRC, circulating miR-17-5p is available to serve as an early biomarker in CRC [15]. MiR-17-5p has also been manifested to exert facilitated effects on CRC progression [16]. Specifically, the cancer-associated fibroblast (CAF)-derived exosomal miR-17-5p promoted CRC malignant phenotypes [17]. Furthermore, high-expressed exosomal miR-17-5p is implicated in pathological stages and grades of CRC patients [18]. These discoveries unraveled the great potential of miR-17-5p in CRC development. As for the programmed death ligand 1 (PD-L1), it has been validated that the administration of anti-PD-L1 contributes to dampening the growth of tumor cells in normal syngeneic mice, suggesting that the PD-L1 serves as an effective modulator for potentially immunogenic tumors to escape from the host immune responses [19]. Relatively, Audrito et al. have elucidated that plasmatic miR-17-5p levels were elevated in metastatic melanoma patients with PD-L1+, validating the marker role of miR-17-5p to PD-L1 expression [20]. In our study, bioinformatics analysis revealed that miR-17-5p had binding sites with Speckle-type POZ (SPOP). SPOP is an E3 ubiquitin ligase adapter that can serve as a tumor suppressor [21]. It has been revealed that silencing SPOP by promoter hypermethylation decreased cell apoptosis in CRC [22], and SPOP has been identified to play a critical role in CRC through mesenchymal–epithelial transition and matrix metalloproteinases [23].

As stated above, miR-17-5p from tumor-derived cells acted as a promising modulator for cancer development and the survival of cancer patients, indicating its potential therapeutic value in cancer treatment; while the detailed function of exosomal miR-17-5p from tumor-derived cells in CRC progression remained obscure. Considering such conditions, we designed the study to explore the function of the CRCSC-derived exosomes (CRCSC-exos) conveying miR-17-5p in CRC development with the involvement of SPOP, and we further speculate that CRCSC-derived exosomal miR-17-7p may target SPOP to regulate the CRC cell growth.

## Results

### CRC stem cell sorting and exosome identification

**Fig. 1:**
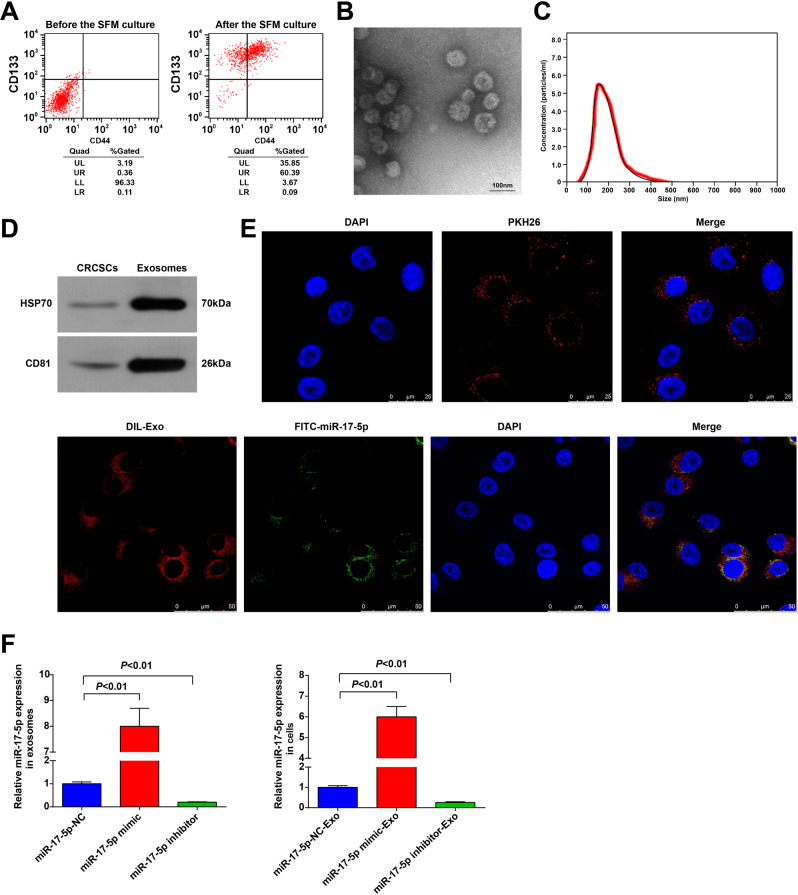
CRC stem cell sorting and exosome identification. **A** CD133^+^CD44^+^ CRCSC were sorted by flow cytometry; **B** the exosome morphology was observed using a TEM; **C** the diameter distribution and concentration of exosomes were detected by NTA; **D** the exosome markers HSP70 and CD81 in CRCSCs and exosomes were detected by western blot analysis; **E** the entry of exosomes that labeled with fluorescent PKH67 to HCT116 cells was observed by immunofluorescence microscopy; the co-localization of labeled fluorescent FITC-miR-17-5p and exosomes in HTC116 cells was observed by immunofluorescence microscopy; FITC-miR-17-5p-labeled exosomes were green; DAPI-stained nuclei were blue; DIL-labeled exosomes were red; **F** miR-17-5p expression in cells and exosomes detected using RT-qPCR. The cell experiments were repeated three times; *P* < 0.05; the measurement data were expressed as mean ± standard deviation.

Surface markers CD133 and CD44 were broadly used to select and isolate the CRC stem cells from CRC cells [24]. In this study, the subpopulation of CD133^+^CD44^+^ accounted for <1.00% in HCT116 cells before the experiment. After incubated in SFM, the ratio of CD133^+^CD44^+^ cells was increased, and these cells were CRC stem cells (Fig. 1A).

As observed through a TEM, the exosomes derived from CD133^+^CD44^+^ HCT116 cells had a membranous morphology (Fig. 1B). Results of NTA implied that exosome diameter ranged from 50 to 300 nm (Fig. 1C). The expression of exosome markers (HSP70 and CD81) could be observed in the exosomes (Fig. 1D). The exosome uptake experiment revealed that the fluorescent PKH26-labeled exosomes successfully entered HCT116 cells. To further explore the transmission of miR-17-5p, we electrotransferred FITC-miR-17-5p (green) into CRCSC, extracted cellular exosomes, added Dil tags (red), and incubated HCT116 cells for 48 h. Co-localization of FITC and Dil was observed in recipient cells, indicating that the FITC-miR-17-5p-containing cellular exosomes were internalized by cells (Fig. 1E). MiR-17-5p was upregulated in exosomes after the CD133^+^CD44^+^ HCT116 cells were introduced with miR-17-5p mimic, and miR-17-5p expression was also increased in HCT116 cells after the cells were co-cultured with exosomes conveying miR-17-5p mimic (Fig. 1F). The results indicated the successful isolation of CRCSC-exos.

### CRCSC-exos promote malignancy of HCT116 cells

As previously reported, the CAF-derived exosomes promoted CRC metastasis [17]. Thus, we speculated that CRCSC-exos may facilitate CRC progression.

**Fig. 2:**
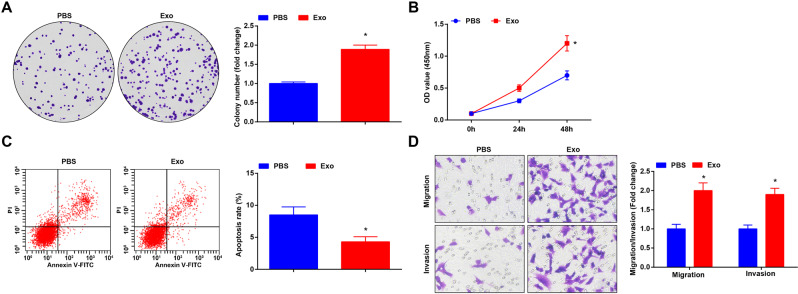
CRCSC-exos promote malignancy of HCT116 cells. **A** Proliferation of HCT116 cells detected using colony formation assay after exosome treatment; **B** proliferation of HCT116 cells detected using CCK-8 assay after exosome treatment; **C** apoptosis of HCT116 cells detected using flow cytometry after exosome treatment; **D** migration and invasion of HCT116 cells detected using Transwell assay (magnification: ×200) after exosome treatment. The cell experiments were repeated three times; *P < 0.05 vs the PBS group; the measurement data were expressed as mean ± standard deviation.

The proliferation was assessed using colony formation assay and CCK-8 assay; apoptosis was determined using flow cytometry; invasion and migration were assessed through Transwell assay. The results of these experiments (Fig. 2A–D) showed that the CRCSC-exos promoted proliferation, migration, and invasion and inhibited apoptosis of HCT116 cells, revealing the oncogenic role of CRCSC-exos in CRC.

### CRCSC-exos reduce anti-tumor immunity in CRC

Generally, the immune system can identify and remove tumor cells in the tumor microenvironment. Nevertheless, tumor cells can affect the expression of T cell cytokines to inhibit the immune system, thus the tumor cell killing was suppressed [25]. It has been reported that PTPN2 deficiency in T cells enhances the efficacy of cancer immuno-monitoring and over-metastatic tumor-specific T cells. T cell-specific PTPN2 deficiency also prevents tumor formation in elderly mice, and tumor suppressor p53 was heterozygous. CD8+ T cells with metastatic PTPN2 defect saliently inhibits the formation of mammary tumor in mice. PTPN2 is a target for enhancing T cell-mediated anti-tumor immunity and CAR-T cell therapy of solid tumors [26]. Such discoveries reflected that the tumor immune microenvironment affected the occurrence and development of tumors, and provided novel insights for tumor treatment.

**Fig. 3:**
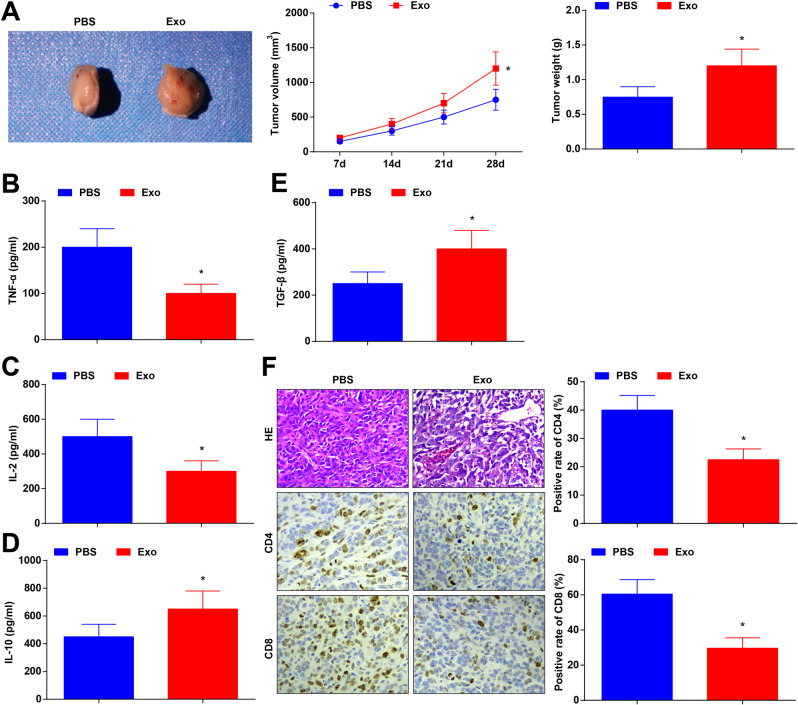
CRCSC-exos reduce anti-tumor immunity in CRC. **A** Representative images, volume, and weight of xenografts from mice after exosome treatment; **B**–**E** TNF-α, IL-2, IL-10, and TGF-β contents in mouse serum were determined using ELISA 72 h after exosome injection; **F** the histological characteristics of tumor tissues detected using HE staining; CD4 and CD8 expression in xenografts from mice detected using immunohistochemistry after exosome treatment. There were five mice in each group; *P < 0.05 vs the PBS group; the measurement data were expressed as mean ± standard deviation.

Here we further explored the effect of CRCSC-exos on the tumor microenvironment by establishing a xenograft mouse model. The results implied that the treatment of CRCSC-exos promoted the tumor volume and weight (Fig. 3A). Serum of experimental mice was collected 72 h after exocrine injection, T cell cytokine contents in mouse serum were determined and it was found that the exosomes reduced tumor necrosis factor-α (TNF-α) and interleukin (IL)-2 contents while increasing IL-10 and transforming growth factor-β (TGF-β) contents in enzyme-linked immunosorbent assay (ELISA; Fig. 3B–E). Moreover, the results of HE staining reflected that the inflammatory infiltration of tumor tissue was promoted after exosome treatment; while the immunohistochemical staining of the tumor section showed that, after exosome treatment, CD4^+^ and CD8^+^ T cell infiltration was alleviated (Fig. 3F).

### CRCSC-exos upregulating miR-17-5p facilitates malignant behaviors of HCT116 cells

**Fig. 4:**
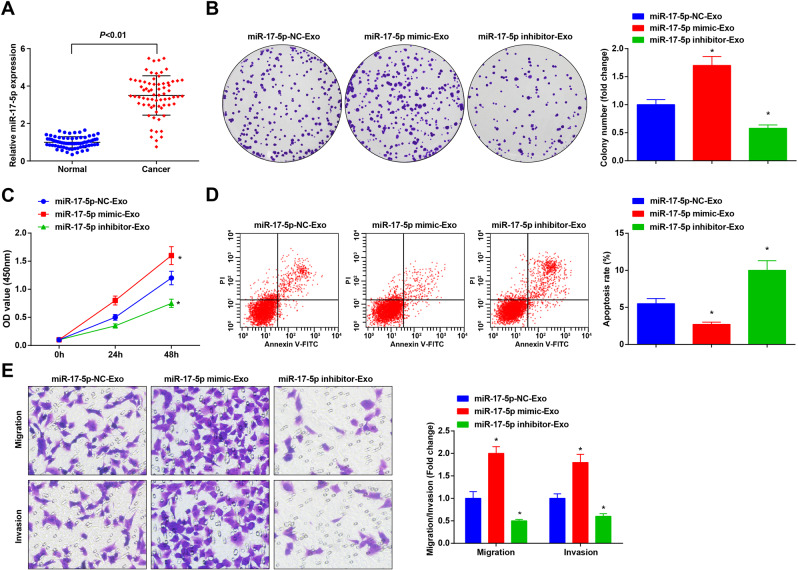
CRCSC-exos upregulating miR-17-5p facilitates malignant behaviors of HCT116 cells. **A** miR-17-5p expression in CRC tissues and adjacent normal tissues detected using RT-qPCR (*n* = 70); **B** proliferation of HCT116 cells detected using colony-formation assay after treated with CRCSC-exos transmitting miR-17-5p mimic/inhibitor; **C** proliferation of HCT116 cells detected using CCK-8 assay after treated with CRCSC-exos transmitting miR-17-5p mimic/inhibitor; **D** apoptosis of HCT116 cells detected using flow cytometry after treated with CRCSC-exos transmitting miR-17-5p mimic/inhibitor; **E** migration and invasion of HCT116 cells detected using Transwell assay after treated with CRCSC-exos transmitting miR-17-5p mimic/inhibitor. The cell experiments were repeated three times; *P < 0.05 vs the miR-17-5p-NC-Exo group; the measurement data were expressed as mean ± standard deviation.

MiR-17-5p participates in CRC progression [17, 27]. Thus, we detected miR-17-5p expression in clinical samples. Our results suggested that miR-17-5p was upregulated in CRC tissues (Fig. 4A).

HCT116 cells were treated with CRCSC-exos transmitting miR-17-5p mimic/inhibitor to explore the role of exosomal miR-17-5p in CRC cell processes. These findings in our experiments reflected that compared to HCT116 cells co-cultured with miR-17-5p-NC-Exo, the exosomal miR-17-5p facilitated proliferation, migration and invasion, and restricted apoptosis of HCT116 cells, while those miR-17-5p inhibitor-Ex treatment exerted opposite effects on HCT116 cells (Fig. 4B–E), indicating the promotive role of upregulated exosomal miR-17-5p in CRC cell growth.

### CRCSC-exos upregulating miR-17-5p suppresses anti-tumor immunity in CRC

**Fig. 5:**
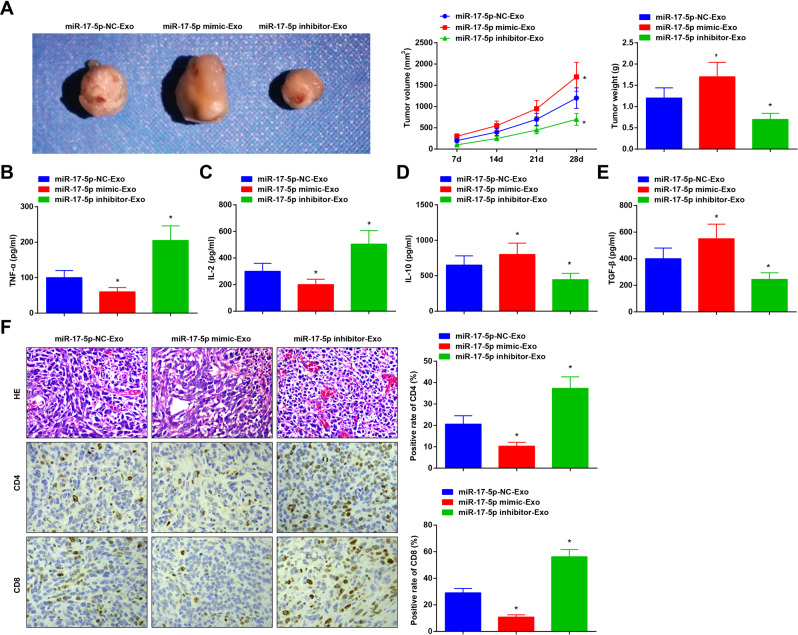
CRCSC-exos upregulating miR-17-5p suppresses anti-tumor immunity in CRC. **A** Representative images, volume, and weight of xenografts from mice after treated with CRCSC-exos transmitting miR-17-5p mimic/inhibitor; **B**–**E** TNF-α, IL-2, IL-10, and TGF-β contents in mouse serum determined using ELISA 72 h after exosome injection; **F** the histological characteristics of tumor tissues detected using HE staining; CD4 and CD8 expression in xenografts from mice detected using immunohistochemistry after treated with CRCSC-exos transmitting miR-17-5p mimic/inhibitor. There were five mice in each group. *P < 0.05 vs the miR-17-5p-NC-Exo group; the measurement data were expressed as mean ± standard deviation.

To unravel the impacts of miR-17-5p on anti-tumor immunity in CRC, the xenograft modeled mice were treated with CRCSC-exos elevating or inhibiting miR-17-5p to reveal the role of exosomal miR-17-5p in anti-tumor immunity in CRC; and tumor samples and mouse serum were tested. The results showed that compared to miR-17-5p-NC-Exo-treated nude mice, CRCSC-exos upregulating miR-17-5p treatment increased tumor volume and weight, reduced serum contents of TNF-α and IL-2, and relieved the CD4^+^ and CD8^+^ T cell infiltration, yet facilitated the inflammatory infiltration and promoted serum contents of IL-10 and TGF-β; the exosomes silencing miR-17-5p treatment exerted contrary effects (Fig. 5A–F). These findings suggested that CRCSC-exos upregulating miR-17-5p deteriorated CRC, promoted tumor growth, and repressed the anti-tumor immunity of CRC.

### MiR-17-5p targets SPOP

**Fig. 6:**
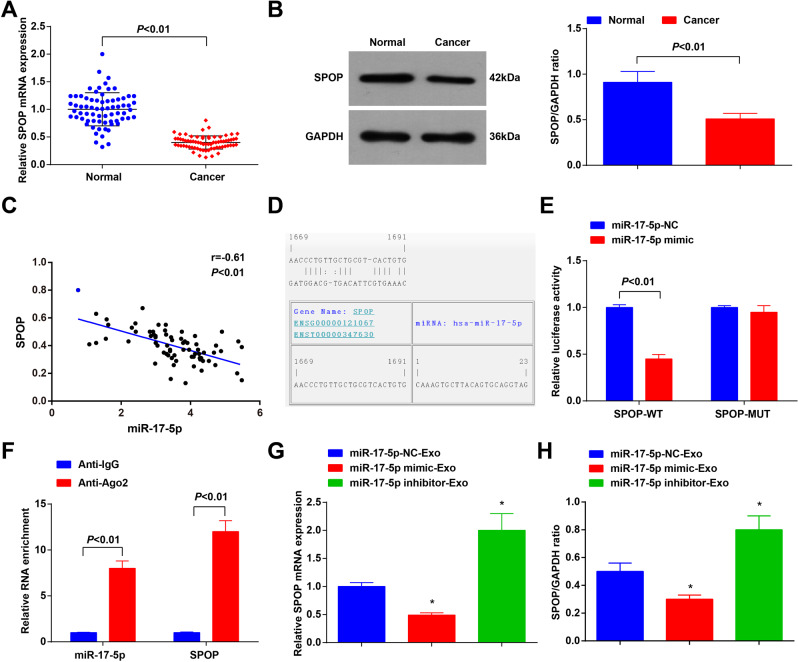
MiR-17-5p targets SPOP. **A** SPOP expression in CRC tissues and adjacent normal tissues detected using RT-qPCR (*n* = 70); **B** SPOP expression in CRC tissues and paracancerous tissues detected using western blot analysis (*n* = 70); **C** correlation between expression of miR-17-5p and SPOP analyzed using Pearson test (*n* = 70); **D** binding sites between miR-17-5p and SPOP predicted at a bioinformatic website; **E** targeting relationship between miR-17-5p and SPOP confirmed using dual-luciferase reporter gene assay; **F** targeting relationship between miR-17-5p and SPOP confirmed using RIP assay; **G** SPOP expression in HCT116 cells detected using RT-qPCR and western blot analysis. Cell experiments were repeated three times; *P < 0.05 vs the miR-17-5p-NC-Exo group; the measurement data were expressed as mean ± standard deviation.

SPOP expression was detected in clinical samples and we found that it was downregulated in CRC (Fig. 6A, B). The result of the Pearson test indicated a negative relationship between the expression of miR-17-5p and SPOP in CRC samples (Fig. 6C). Thus, we speculated that miR-17-5p may target SPOP.

It was predicted at a bioinformatic website that there existed binding sites between miR-17-5p and SPOP (Fig. 6D). The dual-luciferase reporter gene assay further revealed that the co-transfection of SPOP-wt and miR-17-5p mimic suppressed the luciferase activity (Fig. 6E). Moreover, results of RIP assay showed that miR-17-5p and SPOP expression was enriched in Ago2 immunoprecipitation (Fig. 6F). The results of RT-qPCR and Western blot analysis implied that SPOP was downregulated (upregulated) after miR-17-5p expression was increased (decreased) (Fig. 6G). These findings indicated the targeting relationship between miR-17-5p and SPOP.

### SPOP overexpression inhibits biological functions of HCT116 cells

**Fig. 7:**
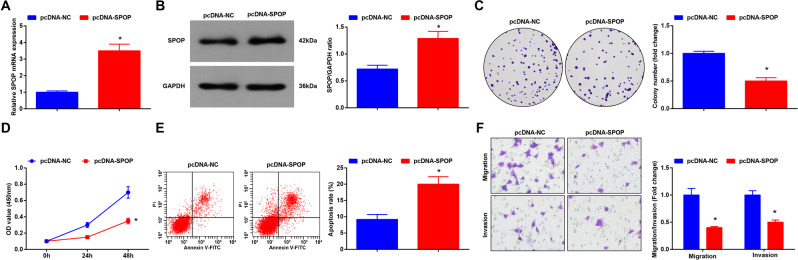
SPOP overexpression inhibits the biological functions of HCT116 cells. **A** SPOP expression in HCT116 cells detected using RT-qPCR after transfected with pcDNA-SPOP; **B** SPOP expression in HCT116 cells detected using western blot analysis after transfected with pcDNA-SPOP; **C** proliferation of HCT116 cells detected using colony formation assay after transfected with pcDNA-SPOP; **D** proliferation of HCT116 cells detected using CCK-8 assay after transfected with pcDNA-SPOP; **E** apoptosis of HCT116 cells detected using flow cytometry after transfected with pcDNA-SPOP; **F** migration and invasion of HCT116 cells detected using Transwell assay after transfected with pcDNA-SPOP. Cell experiments were repeated three times; *P < 0.05 vs the pcDNA-NC group; the measurement data were expressed as mean ± standard deviation.

HCT116 cells were transfected with pcDNA-SPOP to alter SPOP expression, and we found that SPOP expression was successfully increased after the transfection (Fig. 7A, B). Then, the biological functions of transfected HCT116 cells were determined through a series of assays. It was discovered that overexpressed SPOP hindered proliferation, migration, and invasion and promoted apoptosis of HCT116 cells (Fig. 7C–F). Thus, it could be concluded that SPOP upregulation inhibited CRC cell growth.

### MiR-17-5p targets SPOP to upregulate PD-L1, thus affecting the anti-tumor immunity in CRC cells

Immune checkpoint mediated by PD-1 and its ligand PD-L1 has been approved to be used for human cancer treatment, and it has been revealed that SPOP deletion could block the ubiquitin-induced PD-L1 degradation to increase PD-L1 protein expression [28].

**Fig. 8:**
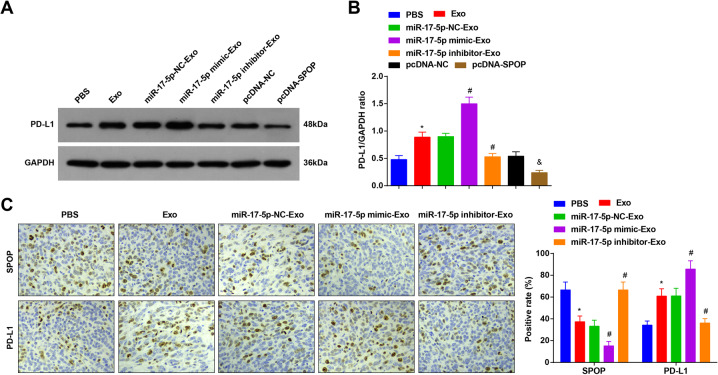
MiR-17-5p targets SPOP to upregulate PD-L1, thus affecting the anti-tumor immunity in CRC cells. **A**, **B** PD-L1 expression in HCT116 expression detected using western blot analysis after treatment with miR-17-5p-mimic/inhibitor- Exo; **C** SPOP and PD-L1 levels examined using immunohistochemistry after treatment with miR-17-5p-mimic/inhibitor -Exo (*n* = 5); cell experiments were repeated three times and there were 5 mice in each group; *P < 0.05 vs the PBS group, ^#^*P* < 0.05 vs the miR-17-5p-NC-Exo group, ^&^*P* < 0.05 vs the pcDNA-NC group; the measurement data were expressed as mean ± standard deviation.

We detected PD-L1 protein expression in HCT116 cells and found that miR-17-5p inhibited SPOP to upregulate PD-L1 (Fig. 8A, B). Immunohistochemical staining detection of SPOP and PD-L1 levels in xenograft tumors showed that miR-17-5p elevated PD-L1 expression by suppressing SPOP expression (Fig. 8C).

## Discussion

CRC is one of the most dangerous cancers and its global incidence and mortality have increased markedly over the past decades. Though genetic and epigenetic changes of CRC have been broadly reported, the molecular mechanisms in the development of CRC remain to be further explored [29]. We aim to investigate the role of CRCSC-derived exosomal miR-17-5p during CRC development with the involvement of SPOP. Our experimental results revealed that CRCSCs-exos upregulated miR-17-5p, which targeted SPOP to promote growth and inhibit anti-tumor immunity in CRC cells through promoting PD-L1.

The effect of exosomes on CRC progression has been reported before. For instance, CAFs-derived exosomes have been validated to promote metastasis and chemotherapy resistance in CRC [30], and Cheng *et al*. have demonstrated that the stem-like tumor exosomes promote tumorigenesis and are associated with an immunosuppressive tumor microenvironment in CRC [31]. In the present study, the CRC cells were transfected with CRCSC-exos to observe the role of the exosomes in CRC cell growth. Our results indicated that the CRCSC-exos promoted proliferation, migration, and invasion and inhibited apoptosis of CRC cells. Moreover, the findings in our in vivo assay suggested that the CRCSC-exos suppressed the tumor growth and anti-tumor immunity in CRC. Similarly, research has uncovered that the exosomes from SW480 CRC cells facilitate the migration of HepG2 hepatocellular cancer cells [32]. Another study has revealed that the increased secretion of CRCSC-exos was accompanied by elevated tumor infiltration of myeloperoxidase neutrophils, which promotes tumorigenesis of CRC cells via IL-1β [24]. These data provided a theoretical basis for identifying the role of CRCSC-exos during CRC development. Additionally, the miRNAs can be secreted into the extracellular space, mostly in the form of exosomes, and function in intercellular communication [8]. It has been suggested that the CRCSC-derived exosomal miR-146a-5p promoted stem-like properties and tumorigenicity in recipient CRC cells [31], indicating the involvement of CRCSC-derived exosomal miRNA in CRC progression. As previously reported, the miR-17-92 cluster shows higher expression in CRC tissues, and only miR-17-5p is differentially expressed among adjacent, adenoma, and CRC tissues among the three miRNAs [33]. Moreover, Ma *et al*. have reported the oncogenic role of miR-17-5p, which is upregulated in CRC tissues and this high expression of miR-17-5p indicates a shorter overall survival rate [16]. Here we detected miR-17-5p expression in clinical samples, and it was found that miR-17-5p was upregulated in CRC tissues versus the adjacent normal tissues. It has been revealed that the CAFs-derived exosomal miR-17-5p promotes the metastasis and progression of CRC [17]. Thus, we speculated that exosomal miR-17-5p may contribute to CRC development. The CRC cells were co-cultured with CRCSC-exos delivering miR-17-5p mimic or inhibitor to verify the role of exosomal miR-17-5p in CRC, and we observed that CRCSC-exos upregulating miR-17-5p facilitated proliferation, migration and invasion, and restricted apoptosis of CRC cells, and also inhibited tumor growth as well as anti-tumor immunity in CRC. Oppositely, CRCSC-exos downregulating miR-17-5p had reverse effects. In addition, we have confirmed the targeting relationship between miR-17-5p and SPOP in CRC cells using bioinformatic prediction and luciferase assay. It is a novel finding that was scarcely explored before. We also detected SPOP expression and it was discovered that SPOP expression was decreased in CRC tissues. Similarly, Xu et al. have reported the frequent SPOP downregulation in CRC tissues versus matched adjacent non-tumorous tissues [23], and a previous study has shown that SPOP expression is remarkably repressed in CRC tissues and indicates poor differentiation status of CRC [34]. The expression of SPOP was regulated in our experiments to identify the role of SPOP in the biological processes of CRC cells. The results in our study suggested that the overexpression of SPOP inhibited malignant behaviors of CRC cells and promoted the anti-tumor immunity in CRC. Consistently, it has been revealed that SPOP serves as a tumor repressor gene in gastric, colorectal, and prostate cancers [35]. Importantly, a publication has reported that the loss-of-function mutation in SPOP compromises ubiquitination-mediated PD-L1 degradation, thus increasing PD-L1 level and reducing the number of tumor-infiltrating lymphocytes in mouse tumors and in clinical prostate cancer specimens [36].

In conclusion, we demonstrated that the elevation of CRCSCs-exosomal miR-17-5p inhibits SPOP to facilitate the growth and restrain the anti-tumor immunity in CRC cells via promoting PD-L1. This study may provide a novel insight into the role of exosomal miR-17-5p in CRC progression. However, more efforts are still needed to reveal the underlying molecular mechanisms.

## Materials and methods

### Study subjects

Seventy CRC patients (26 males and 44 females, aged from 24 years to 84 years; median age: 48 years old) from Shengjing Hospital of China Medical University were recruited. No patients had received any treatment before surgery. Tumor tissues of these patients were collected as the experimental group; while normal tissues 5 cm away from CRC tissues were taken as the control group.

### Cell culture

CRC cell line HCT116 (China Center for Type Culture Collection, Shanghai, China) was incubated with Roswell Park Memorial Institute (RPMI)-1640 medium (Corning Inc., NY, USA) containing 10% fetal bovine serum (FBS) and 1% penicillin–streptomycin.

### CRC stem cell sorting

HCT116 cells were cultured in a sphere formation medium (SFM) as previously described [37]. The cultured cells (1 × 10^7^ cells) were resuspended and incubated with anti-human CD133 (1:10, Miltenyi Biotec GmbH, Bergisch Gladbach, Germany) and anti-CD44 (1:50, eBioscience Inc., CA, USA) for 20 min, and then were analyzed using a flow cytometer. Cells with purity >80% after sorting were used for further analysis. The FACSAria system (Becton-Dickinson, NJ, USA) was used for cell sorting and the analysis was performed using FlowJo software. The sorted CD133^+^CD44^+^HCT116 cells were cultured in SFM.

### Cell transfection

Cells were seeded into 6-well plates 24 h before transfection. When the cell confluence reached about 50%, CD133^+^CD44^+^HCT116 cells were transfected with miR-17-5p-negative control (NC), miR-17-5p mimic, or miR-17-5p inhibitor (RiboBio Co., Ltd., Guangdong, China) using Liposome 3000 (Thermo Fisher Scientific, MA, USA). pcDNA-NC and pcDNA-SPOP plasmids constructed by GenePharma (Shanghai, China) were transfected into HCT116 cells. The medium was replaced 6 h later. After 48-h transfection, cells were collected for follow-up experiments.

### Isolation and identification of CRCSC-exos

CD133^+^CD44^+^ HCT116 cells were centrifuged at 300 × *g* for 10 min to remove dead and floating cells, and then the cells were centrifuged at 2000 × *g* for 10 min to remove cell debris. The supernatant was used to extract the exosomes through the exosome extraction kits (BestBio, Nanjing, China). To ascertain the exosomes identification, transmission electron microscopy (TEM; Hitachi 7700, Tokyo, Japan) was utilized to observe exosome morphology, and nanoparticle tracking analysis (NTA; NanoSight 300; Malvern Instruments, Malvern, UK) was employed to assess exosome size and concentration. Western blot analysis was used to detect exosome marker protein HSP70 and CD81 [17].

### Exosome uptake

PKH26 red fluorescent cell junction Kit (Sigma-Aldrich, CA, USA) was used for cell tracking. The exosomes were resuspended and stained with PKH26 dye solution for 5 min. The same volume of dye solution was added to exosomes immediately and incubated for 5 min. Then, 10% bovine serum albumin (BSA, 600 μL) prepared in Dulbecco’s pluripotent stem cells was added to the exosome for 1-min staining. The 20 μg/mL PKH26-labeled exosomes were incubated with HCT116 cells overnight and the Zeiss LSM880 laser scanning confocal system (Carl Zeiss, Oberkochen, Germany) was used for imaging. The image was processed by ZEN 2009 Light Edition software (Carl Zeiss).

To probe the transmission of miR-17-5p, Fluorescein Isothiocyanate (FITC)-miR-17-5p was packaged into exosomes using electroporation, and isolated from CRCSC culture conditioned medium and packed with FITC-miR-17-5p. Briefly, FITC-miR-17-5p was added to exosomes in electroporation buffer (21% Optiprep, 25 mm KCl, 100 mm potassium phosphate, pH 7.2), and then transferred to a 4 mm electroporation tube (Eppendorf, Hauppauge, NY). Samples were electroporated in the E2510 electroporator (Eppendorf, Hauppauge, NY) with three pulses at 0–2000 V and stored at 4 °C for 5 min. FITC-miR-17-5p (green) was electrotransformed and entered exos. Dil label (red) was added. HCT118 cells were incubated for 48 h. The co-localization of FITC and Dil was observed in recipient cells using a fluorescence microscope (ECLIPSE E800, Nikon, Japan).

### Reverse transcription-quantitative polymerase chain reaction (RT-qPCR)

TRIzol kits (Thermofisher) were used to extract total RNA in tissues and cells, and the mRNA and miRNA were reversely transcribed into cDNA using Prime Script RT Master Mix Kit (TaKaRa, Dalian, China) and Mir-X miRNA First-Strand Synthesis Kit (Takara, Dalian, China). The PCR was performed using the Prime Script RT Master Mix Kit (TaKaRa, Dalian, China). U6 and glyceraldehyde phosphate dehydrogenase (GAPDH) as the endogenous references. Each PCR product was repeated three times. Data were analyzed using the 2^−ΔΔCt^ method [38] and primers (Sangon Biotechnology Co., Ltd., Shanghai, China) are shown in Supplementary Table 1.

### Western blot analysis

Total protein was extracted from tissues and cells. Protein concentrations were measured using a commercial bicinchoninic acid kit (Pierce, Rockford, IL, USA). The extracted protein was added to the loading buffer and boiled at 95 °C for 10 min. There were 30 μg samples in each well. The protein was separated by 10% polyacrylamide gel electrophoresis, the electrophoretic voltage was turned from 80 to 120 V and subjected to wet transfer. The voltage of the transfer membrane was 100 MV, and the time was set for 45–70 min. The protein was removed to polyvinylidene fluoride membrane, blocked with 5% bovine serum albumin for 1 h, and added with primary antibodies GAPDH (1:1000, Sungene, Shanghai, China), SPOP (1:1000, Cell Signaling Technology [CST], MA, USA), PD-L1 (1:1000, CST), HSP70 (1:1000, ABclonal, Wuhan, China) and CD81 (1:1000, ABclonal) at 4 °C overnight. Thereafter, the proteins were rinsed with Tris-buffered saline with Tween 20 for 3 times/5 min, incubated with appropriate secondary antibody (CST, 1:2000) for 1 h, and developed with chemiluminescence reagent. Then, proteins were observed using the ECL Western Blotting Detection system (Millipore Inc., MA, USA). The experiment was repeated 3 times and the average was calculated.

### Colony formation assay

One hundred cells were seeded and the medium was discarded when the colonies could be observed. Colonies were fixed in formaldehyde, stained with 10% Giemsa (Sigma), and counted using a microscope (Leica DMI, Leica Microsystems Inc., IL, USA).

### Cell counting kit-8 (CCK-8) assay

The CCK-8 assay kit (DOJINDO, Shanghai, China) was adopted to test the cell proliferative ability based on the manufacturer’s instructions. Cells were seeded at the 96-well plate at 4000 cells/well and the cell viability at 0, 24, and 48 h was detected using CCK-8 assay based on the manufacturer’s information. The proliferative rate was measured by assessing the absorbance of the cells at 450 nm using a microplate reader (Tecan Group, Ltd., Mannedorf, Switzerland) [39].

### Flow cytometry

The apoptosis rate was examined using the Annexin V-FITC Apoptosis Detection Kit (BioLegend, San Diego, CA, USA). The cells were harvested and resuspended in loading buffer (1 × 10^6^ cells/mL). The cell suspension was then added with 5 μL PI and 5 μL Annexin V -FITC before incubation in a dark chamber for 15 min. The stained cells were analyzed using a BD FACSCalibur cell analyzer (BD Biosciences, NJ, USA). All data were analyzed using the CellQuest software (BD Biosciences) [39].

### Transwell assay

The migration and invasion of cells were assessed using Transwell assay referring to a publication [40]. Specifically, 1 × 10^5^ cells were resuspended in a serum-free medium and plated into the upper transwell chamber (Corning, NY, USA) and cultured in FBS-free medium. A complete growth medium with 10% FBS was added to the lower chamber. After 24 h, the cells remaining on the upper surface of the membrane were discarded with a cotton swab. The invaded and migrated cells were then fixed with 4% paraformaldehyde and stained with 0.5% crystal violet. Thereafter, the cells were imaged using an inverted optical microscope (Olympus Corporation, Tokyo, Japan) and cells in six random visual fields were counted. The experimental procedure of invasion determination was the same as that of the migration test, except that Matrigel (BD Biosciences) was precoated in the upper chamber of transwell during invasion determination [41].

### Establishment of the xenograft mouse model

BALB/C mice aged 5-8 w were injected with 0.5 mL HCT116 cell suspension (~1 × 10^6^ cells) at the subcapsular region of the cecum, and then, respectively, treated after 1 week and divided into 5 groups (*n* = 5): the PBS (injection of PBS), Exo (injection of CRCSC-exos), miR-17-5p-NC-Exo (injection of CRCSC-exos transmitting miR-17-5p NC), miR-17-5p mimic-Exo (injection of CRCSC-exos transmitting miR-17-5p mimic) and miR-17-5p inhibitor-Exo (injection of CRCSC-exos transmitting miR-17-5p inhibitor) groups.

The length and width of tumors were measured using a vernier caliper every 7 days. After the experiment, mice were anesthetized by injection of 0.3% pentobarbital sodium and euthanized with neck dislocation. The xenografts were resected, photographed, and weighed [42].

### Immunohistochemistry

Tumor tissues were fixed with 10% formalin and embedded with paraffin and sliced into 5 µm sections. Sections were then deparaffinized at 60 °C for 1 h and then subjected to xylene, ethanol gradient (100–70%), and water. After incubation with 3% hydrogen peroxide for 30 min, sections were boiled in citrate buffer for 20 min. After being sealed with 5% goat serum, the sections were subjected to incubation with primary antibodies (all from Cell Signaling Technology) anti-CD4 (1:100), anti-CD8ɑ (1:400), anti-SPOP (1:200), anti-PD-L1 (1:200) and then incubated with secondary antibody and SignalStain Boost immunohistochemistry Detection Reagent. Afterward, the sections were subjected to incubation with 3,3’-diaminobenzidine (Vector Laboratories 0), counterstaining with hematoxylin, dehydration with the ethanol gradient (70%–100%) and xylene, and mounting with Permount. The section images were captured through the 3D Histech P250 High Capacity Slide Scanner. The quantification data were collected. Secondary antibodies without primary antibodies were set as NC [43].

### Hematoxylin–eosin (HE) staining

Tumor tissues were collected, fixed in 10% neutral buffered formalin, dehydrated in an alcohol gradient, embedded with paraffin, and cut into 3 μm sections, and then stained with HE. Briefly, the paraffin sections were dewaxed with water and subjected to 1-min staining with hematoxylin, 5-s differentiation, and 2-min staining with eosin. Sections were removed quickly once they became pink. The sections were rinsed with tap water to make the sections indicate obvious blue. The cell nuclei were overtly observed, dehydrated, cleared, and the slides were fixed with neutral gum.

### Enzyme-linked immunosorbent assay

The serum of mice was collected from mice 72 h after injection of exosomes. According to the manufacturer’s instructions of the ELISA kit (ExCell Bio, Shanghai, China), the corresponding reagents and samples were added to assess the absorbance value (A450) of each well. TNF-α, IL-2, IL-10, and TGF-β standard curves (*γ* = 0.9990) were plotted to measure the contents of T cell cytokines in mouse serum [42].

### Dual-luciferase reporter gene assay

Binding sites between miR-17-5p and SPOP were predicted at a bioinformatics website. The 3′-UTR sequence of wild-type (WT) or mutant-type (Mut) SPOP was inserted into the pmirGLO Dual-Luciferase vector (Promega, Madison, WI, USA). The SPOP-wild type (wt) and SPOP-mutant type (mut) were established and respectively co-transfected with miR-17-5p-mimic and miR-17-5p-NC into HCT116 cells for 48 h. Cells were lysed and the luciferase activity was assessed.

### RNA immunoprecipitation (RIP) assay

The RIP assay was performed using Magna RNA kits (Millipore). HCT116 cells were lysed using RIP buffer solution and incubated with RIP buffer solution containing magnetic beads conjugated with Ago2 (1:1000, Abcam Inc., MA, USA) and immunoglobulin G (IgG, 1:200, Santa Cruz Biotechnology, CA, USA).

### Statistical analysis

All data analyses were conducted using the SPSS 21.0 software (IBM Corp. Armonk, NY, USA). The measurement data were expressed as mean ± standard deviation. The *t* test was performed for comparisons between two groups and analysis of variance was used for comparisons among multiple groups, followed by Tukey's post hoc test. *P* value <0.05 was indicative of a statistically significant difference.

## Supplementary information

Supplementary Table 1

aj-checklist

original figures for blots

## Data Availability

The original contributions presented in the study are included in the article/Supplementary Material, and further inquiries can be directed to the corresponding author.
