# Supplementary material for: Tumor stem cell-derived exosomal microRNA-17-5p inhibits anti-tumor immunity in colorectal cancer via targeting SPOP and overexpressing PD-L1
Source: Cell Death Discov. 2022 Apr 23;8:223. doi: 10.1038/s41420-022-00919-4 (PMC9035163; doi:10.1038/s41420-022-00919-4)
Supplement: Supplementary file 1 — Supplementary Table 1 [file 41420_2022_919_MOESM1_ESM.docx]

**Supplementary Table 1** Primer sequences for genes in PCR assay

| Gene | Sequence (5’→3’) |
| --- | --- |
| miR-17-5p | F: CAAAGTGCTTACAGTGCAGGTAG |
|  | R: Universal primer |
| SPOP | F: GGAAGGCTCCAAACCTCGACAA |
|  | R: AGCGTTCTCCACGGACAGGTTA |
| U6 | F: ATTGGAACGATACAGAGAAGATT |
|  | R: GGAACGCTTCACGAATTTG |
| GAPDH | F: CGGAGTCAACGGATTTGGTCGTAT |
|  | R: AGCCTTCTCCATGGTGGTGAAGAC |

Note: F, forward; R, reverse; MiR-17-5p, microRNA-17-5p; GAPDH, glyceraldehyde phosphate dehydrogenase.
