# Supplementary material for: Tumor stem cell-derived exosomal microRNA-17-5p inhibits anti-tumor immunity in colorectal cancer via targeting SPOP and overexpressing PD-L1
Source: Cell Death Discov. 2022 Apr 23;8:223. doi: 10.1038/s41420-022-00919-4 (PMC9035163; doi:10.1038/s41420-022-00919-4)
Supplement: Supplementary file 3 — original figures for blots [file 41420_2022_919_MOESM3_ESM.docx]

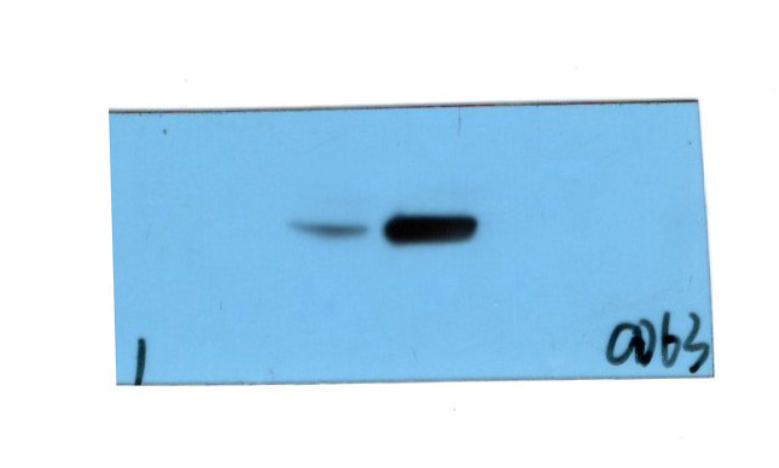

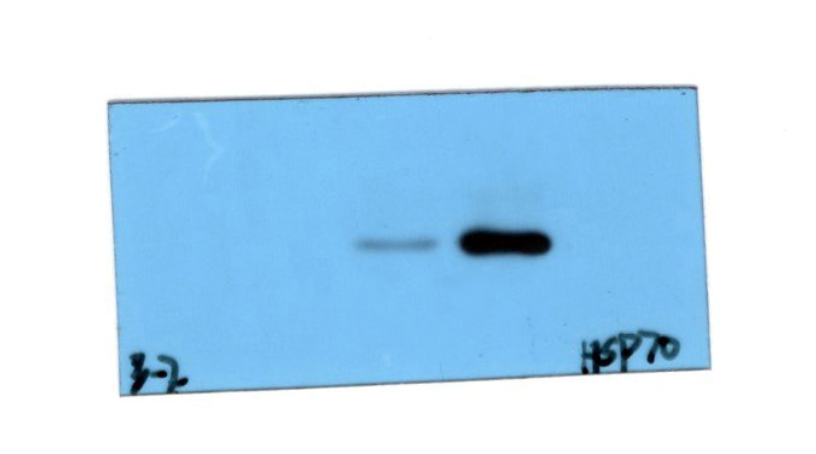


Original figures for blots in Figure 1D


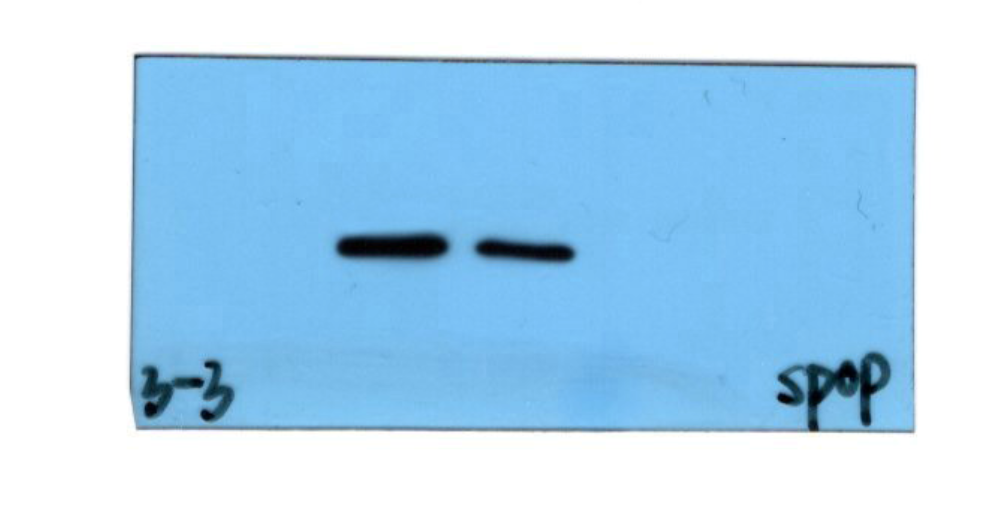


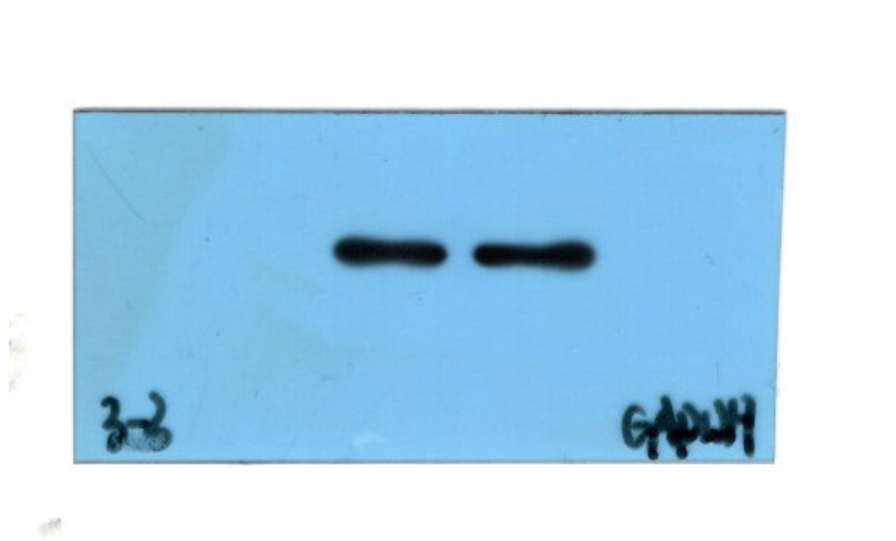


Original figures for blots in Figure 6B


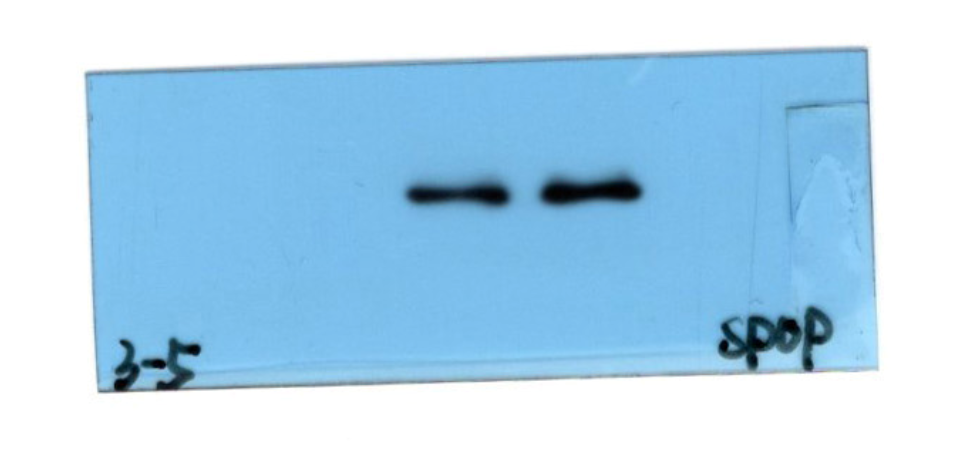

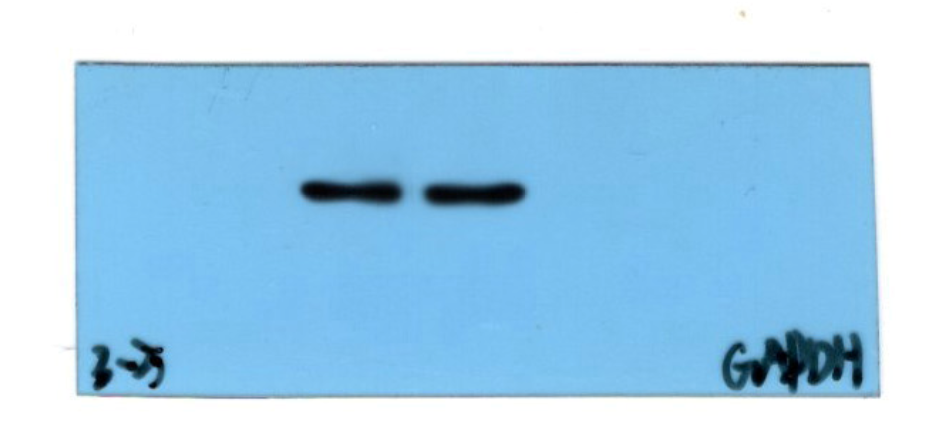


Original figures for blots in Figure 7B


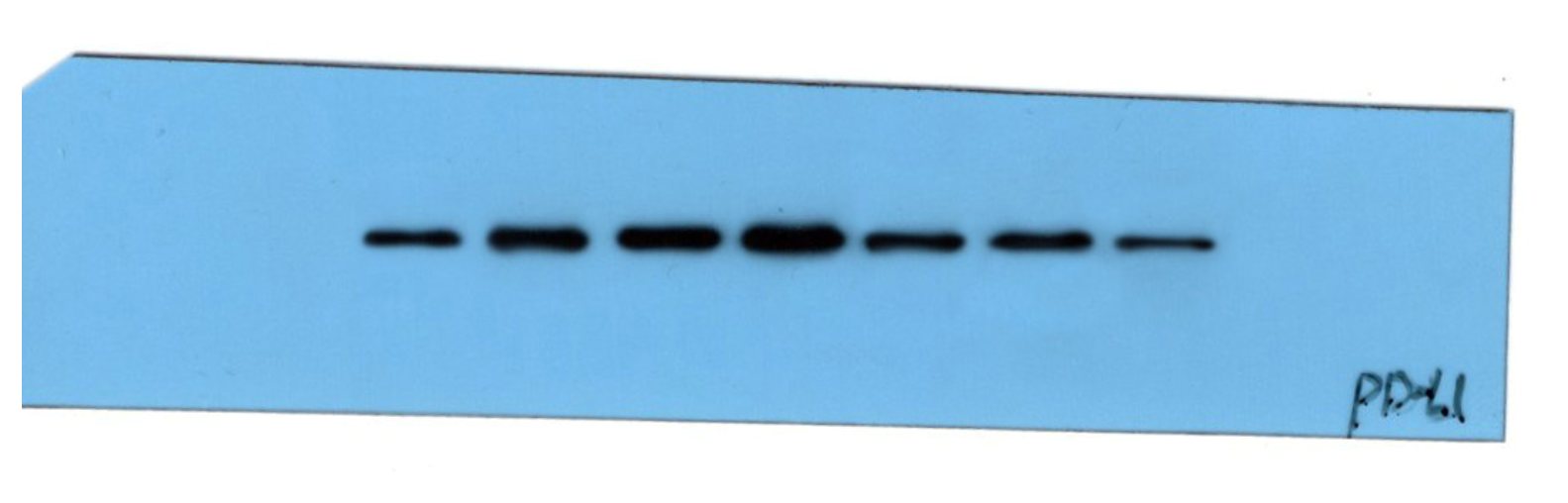

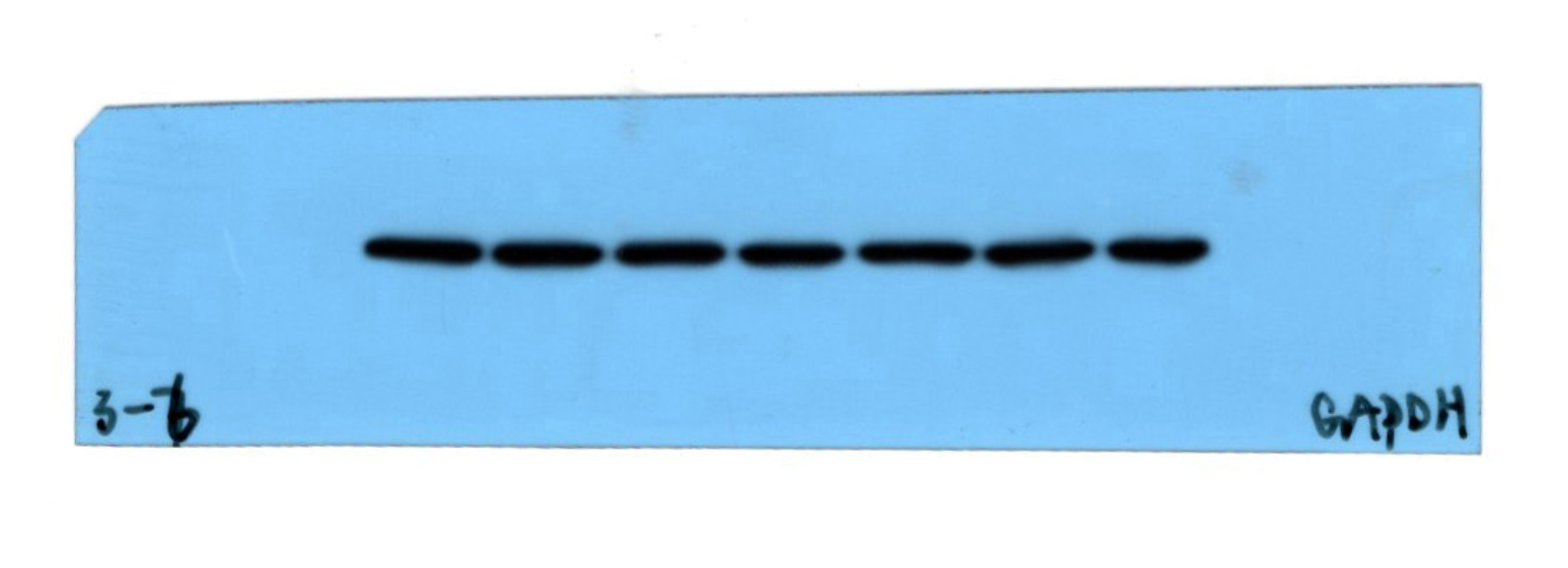


Original figures for blots in Figure 8A
